# Nanostructure and mechanics of mummified type I collagen from the 5300-year-old Tyrolean Iceman

**DOI:** 10.1098/rspb.2010.0377

**Published:** 2010-03-31

**Authors:** Marek Janko, Albert Zink, Alexander M. Gigler, Wolfgang M. Heckl, Robert W. Stark

**Affiliations:** 1 Department of Earth and Environmental Sciences, Ludwig-Maximilians-Universität München, Theresienstraße 41, 80333 Munich, Germany; 2 Center for NanoSciences, Ludwig-Maximilians-Universität München, Schellingstraße 4, 80799 Munich, Germany; 3 European Academy of Bolzano, Institute for Mummies and the Iceman, Viale Druso 1, 39100 Bolzano, Italy; 4 Deutsches Museum, Museumsinsel 1, 80538 Munich, Germany

**Keywords:** ancient collagen, degradation, atomic force microscopy, nanoindentation, Raman spectroscopy, Iceman

## Abstract

Skin protects the body from pathogens and degradation. Mummified skin in particular is extremely resistant to decomposition. External influences or the action of micro-organisms, however, can degrade the connective tissue and lay the subjacent tissue open. To determine the degree of tissue preservation in mummified human skin and, in particular, the reason for its durability, we investigated the structural integrity of its main protein, type I collagen. We extracted samples from the Neolithic glacier mummy known as ‘the Iceman’. Atomic force microscopy (AFM) revealed collagen fibrils that had characteristic banding patterns of 69 ± 5 nm periodicity. Both the microstructure and the ultrastructure of dermal collagen bundles and fibrils were largely unaltered and extremely well preserved by the natural conservation process. Raman spectra of the ancient collagen indicated that there were no significant modifications in the molecular structure. However, AFM nanoindentation measurements showed slight changes in the mechanical behaviour of the fibrils. Young's modulus of single mummified fibrils was 4.1 ± 1.1 GPa, whereas the elasticity of recent collagen averages 3.2 ± 1.0 GPa. The excellent preservation of the collagen indicates that dehydration owing to freeze-drying of the collagen is the main process in mummification and that the influence of the degradation processes can be addressed, even after 5300 years.

## 1. Introduction

Skin is the anatomical outer shielding of the body. Even when mummified, it guards the underlying tissue from external influences and degradation. Its function, however, persists only so long as the skin is intact. Temperature variations, ultraviolet (UV) irradiation and the actions of insects, bacteria and fungi can all cause abrasion and degradation of the skin, enabling biological agents to cross this barrier and causing further tissue decay. The main biomolecular scaffold of skin is type I collagen. It is the most abundant collagen of the human body, and it is also present in scar tissue, interstitial tissue, tendons, arterial walls, fibrocartilage and as an organic constituent of bone (Van der Rest & Garrone 1991). Apart from its high tensile strength and elasticity, type I collagen is known for its exceptional durability. Remnants have even been found in prehistoric samples, such as in the fossilized bones of *Tyrannosaurus rex* (Schweitzer *et al*. 2007*a*) or the fossilized skin of *Psittacosaurus* (Lingham-Soliar 2008). In these specimens, the preservation of collagen is facilitated by its sequestration within the bone or by the mineralization of the soft organic tissue (Schweitzer *et al*. 2007*b*).

Excellent preservation of collagen has been reported for mummified human tissue. Optical and scanning electron microscopy (transmission electron microscopy (TEM)/scanning electron microscopy (SEM)) investigations have revealed single fibrils and bundles of type I collagen in naturally mummified bodies (Williams *et al*. 1995; Hess *et al*. 1998; Stücker *et al*. 2001; Shin *et al*. 2003; Chang *et al*. 2006) and in artificially embalmed mummies (Hino *et al*. 1982; Montes *et al*. 1985). However, the reason for the durability of mummified collagen is unclear. In this paper, we have investigated the structural integrity of mummified collagen in the oldest known glacier mummy, i.e. the *ca* 5300-year-old Tyrolean Iceman. This will help to determine the cause for the stability of mummified collagen and yield a clearer understanding of the mummification process.

The Iceman is a remarkably well-preserved wet mummy and was mummified naturally by a form of freeze-drying. The body was found at 3200 m above sea level, partly embedded in glacier ice. SEM studies of the mummy tissue showed that the collagen fibrils in the skin were structurally preserved (Williams *et al*. 1995; Hess *et al*. 1998). Nonetheless, neither the degree of tissue preservation nor the reason for the preservation of the ultrastructure and molecular structure of the mummified connective tissue is known. The molecular process that led to the outstanding preservation of the Iceman remains unclear.

In this study, we performed atomic force microscope (AFM) measurements and observed typical collagen fibril assemblies in the skin of the Iceman. Complementary confocal Raman spectroscopy measurements support the observation that collagen is preserved by the freeze-drying mummification process. Because nanoindentation measurements by AFM provide access to the nanomechanical properties of collagen at the molecular level (Fratzl *et al*. 1998; Strasser *et al*. 2007; Yang *et al*. 2008), we conducted such experiments on mummified and recent collagen. The resulting data show that Young's modulus of ancient type I collagen is slightly different from that of a recent sample.

## 2. Material and methods

### (a) Collagen preparation

**Figure 1.**
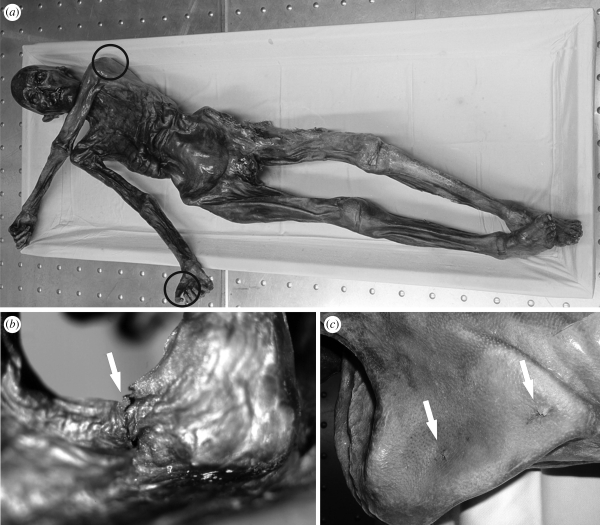
(*a*) The Neolithic glacier mummy, the Iceman. (*b*) Detail of a stab trauma on the right hand of the mummy (sample A). Samples B and C were taken from a haematoma close to the vertebral column and from a wound under the left shoulder blade (spina scapulae), at the Iceman's back (*c*). Photos courtesy of M. Samadelli, South Tyrol Museum of Archaeology, Bolzano, Italy.

Three 5 × 5 mm skin samples were taken from the mummy by punch biopsies. For this purpose, the mummified body (which is stored in a cooling chamber at −6°C and a relative humidity of 98%) was thawed slightly. One specimen was derived from a stab trauma on the right hand (sample A; Nerlich *et al*. 2003). A second sample was taken from the back of the mummy close to the vertebral column (sample B) and the third sample was drawn from a wound under the left *spina scapulae*, at the back of the Iceman (sample C; figure 1). This skin wound is assumed to be the intravital point of an arrowhead entry (Gostner & Vigl 2002; Pernter *et al*. 2007). After extraction, the samples were slowly rehydrated and then fixed and embedded in paraffin wax (Nerlich *et al*. 2003). To obtain histological specimens, 2–4 µm-thick transverse sections were cut and transferred onto glass slides. Before AFM analysis, the paraffin was dissolved in xylene. Subsequently, the sections were rehydrated with a descending alcohol series, rinsed with ultrapure water and dried under ambient conditions. As a reference, we used a comparable recent human skin sample that was taken from a volunteer. To prepare the histological section, the recent skin sample was subjected to the same processes as the mummified tissue, except that the rehydration step before fixation was omitted. After processing, all samples were stored at room temperature in coverslip boxes.

### (b) AFM measurements

The tissue contents and arrangements were inspected using an inverted optical microscope (Axiovert 135, Zeiss, Oberkochen, Germany), and the appropriate sample areas for AFM imaging were defined. The AFM measurements were performed using a NanoWizard-II (JPK Instruments, Berlin, Germany). The AFM was operated under ambient conditions in the intermittent contact mode. Silicon cantilevers (BS Tap 300, Budget Sensors, Redding, USA) with typical spring constants of 40 N m^−1^ and nominal resonance frequencies of 300 kHz were used. The nominal tip radius was smaller than 10 nm. The images were analysed using SPIP (SPIP 4.5.2, Image Metrology, Hørsholm, Denmark). In addition, we examined the mechanical properties of single collagen fibrils using AFM nanoindentation measurements. The Young's moduli of the ancient and the recent collagen samples were determined by a fitting procedure. Both datasets were analysed with the independent two-sample Student's *t*-test. Differences were considered as statistically significant for *p* < 0.01. A detailed description of the measurements and the calculation of Young's modulus are given in appendix A.

### (c) Raman spectroscopy

Confocal Raman spectroscopic measurements were performed using a WITec alpha 300 R microscope (WITec GmbH, Ulm, Germany). The excitation wavelength was 532 nm. The laser power was limited to 1.0 mW to avoid tissue damage. The spectrometer was operated with 600 and 1800 g mm^−1^ gratings. We analysed the wavenumber range from 600 to 3600 cm^−1^ or from 840 to 1900 cm^−1^. The spectral resolution was 3 cm^−1^ per CCD-pixel for the survey spectra and 1 cm^−1^ per CCD-pixel for the high-resolution spectra. For each sample, three different positions were analysed, and at least three single spectra were taken at each position to exclude external effects. The spectra were integrated for 240 s each.

## 3. Results

### (a) AFM imaging—structural preservation of collagen

**Figure 2.**
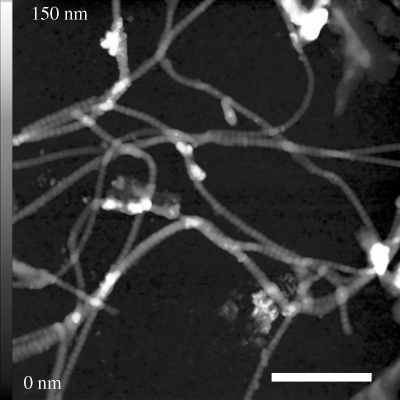
An AFM topography image with a scan size of 4 × 4 µm. A meshwork of randomly oriented single collagen fibrils within sample A can be seen in the figure. Scale bar, 1 µm.

**Figure 3.**
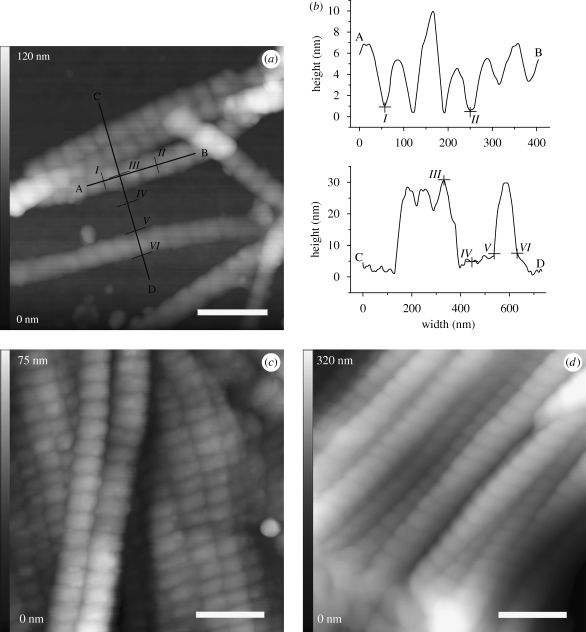
High-resolution images of individual collagen fibrils within Iceman sample A (*a*), showing fibrils stacked in sheet-like structures as observed for Iceman samples B and C (*c*,*d*). Each fibril shows the periodic banding pattern. For sample A, typical fibril profiles that were measured along line AB, perpendicular to the fibril axes (line CD), are shown in (*b*). The distance between sequential bands (e.g. from *I* to *II*) was approximately 68 nm. The height (*III* and *IV*) averaged to 32 nm and the fibril width (*V* and *VI*) was approximately 90 nm. Scale bars, (*a*,*c*,*d*) 250 nm.

We observed single collagen fibrils and fibrils stacked to sheet-like structures within all histological tissue samples. Networks of extremely well-preserved collagen were found, and single fibrils were unsorted and partly overlapping at some sites (figure 2). The fibrils were without any evidence of breaks or fragmentation. Higher magnification also revealed periodic banding patterns (figure 3). For sample A (figure 3*a*,*b*), the topographic analysis along the longitudinal axis of several fibrils indicates a mean banding pattern of 68.3 nm (±5.9 nm s.d.). Measuring the dimensions perpendicular to the longitudinal axis yielded a fibril width of 89.5 ± 5.7 nm and a height of 32.3 ± 5.0 nm. The AFM images of samples B and C showed similar collagen structures. We observed both single collagen fibrils arranged in a meshwork and stacked structures of collagen that formed sheet-like structures (figure 3*c*,*d*). For sample B, the average D-period derived from the banding patterns of more than 60 different fibrils was 69.2 ± 4.9 nm. The fibrils from sample C showed a mean banding pattern of 68.9 ± 4.5 nm. Additional AFM data has been represented as electronic supplementary material.

### (b) Raman spectroscopy—molecular preservation of collagen

**Figure 4.**
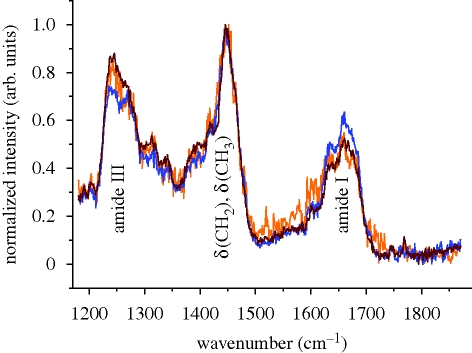
Raman spectra (1170–1870 cm^−1^) of type I collagen acquired on Iceman samples A–C. Each spectrum is averaged from at least three single spectra, taken with integration times of 240 s at three different sample positions. The spectra are normalized to the intensity of the 1448 cm^−1^ band, which was assigned to methyl δ(CH_3_) and methylene δ(CH_2_) groups. Apart from minor differences in the intensities of the bands and the noise level, the spectra are similar. Orange line, Iceman sample A, blue line, Iceman sample B, brown line, Iceman sample C.

Raman spectroscopy is sensitive to both the chemical structure and the conformation of molecules. Spectra were acquired from all three Iceman samples by confocal Raman spectroscopy. The spectra are compared in the region from 1170 to 1870 cm^−1^ in figure 4. The strong line at 1448 cm^−1^ is the deformation vibration of methyl δ(CH_3_) and methylene δ(CH_2_) molecules that are present in proteins (Gniadecka *et al*. 1998; Jastrzebska *et al*. 2005). All of the spectra were normalized to this peak. At approximately 1240 and 1270 cm^−1^, deformation bands of amide III groups appear. They are related to the C–N stretching ν(CN) and N–H in-plane deformation δ(NH) modes (Frushour & Koenig 1975). From 1300 to 1400 cm^−1^, the prominent bands that occur arise from vibrational modes of methylene. In this region, the samples show peaks at 1315, 1339 and 1396 cm^−1^. These correspond to the twisting γ_t_(CH_2_), wagging γ_w_(CH_2_) and deformation δ(CH_2_) modes of methylene, respectively (Frushour & Koenig 1975; Edwards *et al*. 1997). The Iceman samples feature a shoulder at 1421 cm^−1^, which is associated with a stretching mode of COO^−^ (Frushour & Koenig 1975). The shoulders at 1583 and 1604 cm^−1^ can be assigned to the aromatic ring stretch modes ν(CCH) of the amino acids proline/hydroxyproline and tyrosine/phenylalanine, respectively (Frushour & Koenig 1975; Edwards *et al*. 1997). At approximately 1640 cm^−1^ a shoulder is present, and at 1664 cm^−1^ a strong band occurs. These correspond to the C=C stretching vibration ν(CC) and the C=O stretching vibration ν(CO) of amide I groups (Frushour & Koenig 1975). The spectra are consistent except in their intensities and noise levels.

**Figure 5.**
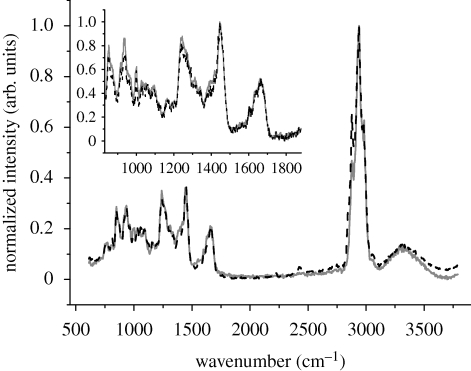
Average Raman spectrum (600–3600 cm^−1^) of recent human skin collagen (dashed line) and of Iceman sample C (solid line). The spectrum of the mummy collagen is similar to the recent collagen spectrum. The inset shows a close-up from 840 to 1900 cm^−1^ wavenumbers, and the spectra have been normalized to the 1448 cm^−1^ peak.

Comparison of the ancient and the recent spectra of type I collagen samples reveals similar features. Representative Raman spectra (600–3600 cm^−1^) for sample C and the recent collagen are shown in figure 5. The strongest band occurs in the range from 2800 to 3050 cm^−1^. This band is associated with the C–H vibrational modes ν(CH_2_) and ν(CH_3_) (Edwards *et al*. 1997). The spectra were normalized to this band. From 3000 to 3800 cm^−1^, the characteristic O–H vibrations of water appear (Jastrzebska *et al*. 2005). The fingerprint region of collagen, showing the amide I and amide III peaks, is presented as the inset in figure 5. No spectral differences were observed.

### (c) AFM nanoindentation—mechanical properties of mummified collagen

**Figure 6.**
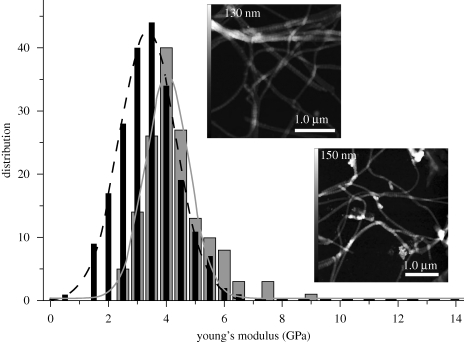
The distribution of Young's moduli measured from mummified and contemporary single human collagen fibrils. A total number of *n* ≥ 150 force–distance curves were evaluated for each sample. The nanoindentation measurement indicates a higher Young's modulus for the mummified human collagen (solid line) compared with the contemporary collagen (dashed line). The upper (recent) and lower (ancient) insets are AFM images showing typical fibrils. Force–distance curves were only recorded from well-separated single fibrils.

To assess the elasticity of the collagen fibrils, we conducted AFM nanoindentation experiments. The numerical value for Young's modulus was obtained as described in appendix A. Force curves that were obtained from the samples of mummified collagen featured a steeper loading-slope than that of the recent collagen. Loading forces of 25 nN caused a 0.7 nm fibril indentation in the recent collagen. By contrast, the mummified collagen was indented by only 0.5 nm using the same forces. As shown in figure 6, Young's modulus of the mummified collagen from Iceman sample A (grey columns) exceeded Young's modulus of the reference sample (black columns). A Gaussian distribution was fitted to each histogram. The upper inset in figure 6 shows the scope of the analysed collagen fibrils, as distributed in the recent skin sample. Single fibrils are oriented randomly and sometimes overlap in multifibril structures. The lower inset displays similar entangled fibril assemblies that were analysed from the Iceman sample. For the ancient collagen that was extracted from the stab wound on the right hand of the mummy, we analysed a total number of 150 force–distance curves. The measurements yielded a mean value of 4.1 ± 1.1 GPa, and the distribution maximum was 4 GPa. For the recent collagen sample, we evaluated 213 curves. The elasticity of the fibrils averaged 3.2 ± 1.0 GPa. The distribution maximum was 3.5 GPa, and 55.4 per cent of the measured data were between 3.0 and 4.0 GPa. The difference between the mean Young's modulus of the ancient and the recent collagen was statistically significant. The scatter of the measured elasticity values can be attributed to anisotropies in the collagen fibril structure. Nanoindentation experiments by Wenger *et al*. (2007) showed that the anisotropic mechanical properties of the fibrils are owing to the tropocollagen subfibrils and their lateral displacement along the fibril axes.

## 4. Discussion

The structure of the tropocollagen triple helix is stabilized by the formation of one interchain hydrogen bond per sequence between the N–H groups of glycines and the C=O groups of prolines on the neighbouring chain (Rich & Crick 1955; Fraser *et al*. 1979). Staggered, parallel tropocollagen molecules covalently cross-link with each other through their aldehyde and amino groups, forming collagen fibrils (Buehler 2006). These self-assembled fibrils feature a characteristic 67 nm, D-periodic banding pattern (Williams *et al*. 1978; Orgel *et al*. 2006). Collagen self-organizes to form bundles or a meshwork that determines the tensile strength, the elasticity and the geometry of the tissue. Enzymes such as collagenase cleave the collagen and are used by micro-organisms to invade the host by degrading the native collagen in the connective tissue (Lecroisey & Keil 1979). By unfolding the triple helical conformation of collagen, the protein is denatured (Davis *et al*. 2000). Subsequently, putrefaction metabolizes the connective tissue into gases, liquids and protein debris. This degradation, caused by the attack of bacteria and fungi, is also a major factor opposing the preservation of mummified tissue.

In our study, morphologically intact type I collagen was identified by topographic analysis for all samples that were extracted from the mummy. The collagen was arranged as single fibrils in a meshwork or stacked in sheet-like structures, as is characteristic for recent skin collagen. The fibrils were undamaged and without any evidence of degradation. The D-period values agree with those found in the literature, i.e. a 67 nm axial repeat (Van der Rest & Garrone 1991; Holmes *et al*. 2001; Orgel *et al*. 2006). Measurements on recent human collagen type I revealed similar values, excluding an effect of the tissue type on variations of the D-period. The mean fibril height (32 nm) corresponds to the value reported for type I collagen (Holmes *et al*. 2001). The width of the fibrils was affected by the dilation of the cylindrical shape of the fibril and the geometry of the AFM tip. Aqueous media possibly influence the fibril diameter, as discussed, for example, by Kato *et al*. (2001) and Stücker *et al*. (2001), but this does not affect the comparison between the ancient and recent tissue, because all histological samples were processed following the same protocol and stored under the same conditions.

Our results indicate that the type I collagen fibrils were preserved through the ages in the mummified skin of the Iceman. This observation is supported by earlier SEM studies, where the preservation of collagen has been documented for other mummies. In SEM studies of 1600 to 2300-year-old bog bodies, dense collagen bundles were observed. Within the bundles, single fibrils with a banding periodicity of 64 ± 3 nm were found (Stücker *et al*. 2001). Chang *et al*. (2006) found similar histological patterns in a well-preserved medieval mummy that was dated from AD 1418 to AD 1450. Good structural preservation of collagen was also indicated in SEM data obtained using a specimen taken from the Iceman. Hess *et al*. (1998) imaged collagen fibrils within the *stratum fibrosum* of the rib bone of the Iceman by TEM and found that structures which were distant from the sites of enzymatic activity were not affected by autolysis. They identified collagen that had an ultrastructural periodicity of 64–67 nm. Williams *et al*. (1995) showed that the distinct skin layers of the dermis, the nucleate epidermis and the outermost skin layer—the *stratum corneum*—of the Iceman persisted over 5300 years.

By investigating collagen from wound tissue, we go a step further. Although some samples were drawn from wounds where the tissue is typically prone to degradation, the submicron structure of skin from the Iceman revealed the same features as those observed in undamaged specimens. We did not detect fibril debris or residues from degradation. From the AFM measurements, we conclude that the ultrastructure of the collagen fibrils was not altered. Based on these observations, we can exclude the decomposition and alteration of the fibril structure by insect or micro-organism infestation.

This conclusion is also confirmed by Raman measurements. Within the spectra taken from the Iceman samples, we detected bands that are characteristic of type I collagen. The presence of the amide I (1667 cm^−1^) and amide III (1245–1270 cm^−1^) bands, which represent the peptide bonds within proteins, points to a helical conformation of the collagen molecules and, hence, an intact collagen structure (Gross *et al*. 1954; Gross 1956; Frushour & Koenig 1975). In addition, we can assume that there were no molecular alterations in the collagen, because the positions of the Raman bands in the Iceman samples were similar to those found in the recent samples. Furthermore, the spectra of the Iceman samples were as distinct as the spectra of recent samples. The spectrum of sample A taken from the Iceman was slightly noisier than the spectra of samples B and C (figure 4). This is because the peak intensities in the Raman spectra of samples B and C were higher, yielding a lower signal to noise ratio for sample A. These variations in intensity may arise from different thicknesses of the samples and a different adjustment of the laser focus. A superposition of the Raman signal of collagen with the Raman signal of the underlying glass substrate does not affect the spectral region between 1170 and 870 cm^−1^. Similarly, a comparison of the Raman spectra at higher wavenumbers shows that the protein molecules are preserved. The bands assigned to the C–H vibrational modes between 2800 and 3050 cm^−1^ and the O–H vibrational modes from 3000 to 3800 cm^−1^ are identical for ancient collagen and the spectrum from recent human skin.

Nanoindentation measurements, however, revealed small changes in the mechanical behaviour of the fibrils. Young's modulus of the mummified collagen was 4.1 ± 1.1 GPa, whereas the collagen of the contemporary skin sample was 3.2 ± 1.0 GPa. This observation indicates that some of the collagen was altered slightly. Changes in the mechanical properties of the ancient collagen may have occurred owing to freeze–thaw cycles, irradiation by UV light, or dehydration of the tissue. Freeze–thaw damage can change the molecular structure of collagen, as the crystallization of the ice can disrupt and break the fibrils. This would result in an altered Young's modulus and a distortion or fragmentation of the collagen. Nevertheless, no distortion, fragmentation or changes in the molecular structure of the collagen were observed.

The effect of UV irradiation on collagen molecules is still under debate. The impact of UV irradiation depends mainly on the time, dose and wavelength of the irradiation and on the environmental conditions. Short irradiation of collagen stored in water, or under nitrogen atmosphere increases its stability (Weadock *et al*. 1995; Sionkowska 2005). However, under ambient conditions and for longer irradiation times, the stability decreases and the mechanical stiffness is reduced. The loss of stability can be attributed to scission, fragmentation and denaturation of the collagen polypeptide chains (Miyata *et al*. 1971; Weadock *et al*. 1995; Ohan *et al*. 2002; Sionkowska 2005). Nonetheless, the impact of UV irradiation on the mummified collagen has to be extrapolated from the level of tissue preservation, because the environmental exposure of the Iceman over the past 5300 years is unknown. Our nanoindentation measurements suggest that there have been only short periods of UV irradiation of the body because Young's modulus for the ancient collagen was increased slightly.

Dehydration and the formation of additional cross-links appear to be the major factor responsible for alterations of the mechanical properties. Removal of the interstitial water can bring the collagen subfibrils closer together, enabling the formation of additional cross-links. Kato *et al*. (2001) have shown that this leads to an increase in fibril stiffness. This effect was also observed in molecular dynamics simulations, where the absence of water in collagen-like peptides caused a distortion of the molecular conformation and, simultaneously, induced additional intra-molecular hydrogen bonds (Mogilner *et al*. 2002). Recent proliferation experiments suggest that dehydrated collagen fibrils become mechanically stiffer than fully hydrated fibrils, indicating that the level of H-bonding is increased (McDaniel *et al*. 2007). Our experimental data shows an increase of the stiffness from 3.2 GPa for the recent collagen to 4.1 GPa for the mummy collagen. We suggest that the strong dehydration of the mummy tissue led to the formation of additional interpeptide H-bonds or covalent cross-links (Buehler 2008) between the tropocollagen subfibrils.

Additional cross-linking owing to the advanced glycation endproduct (AGE) formation (Cerami *et al*. 1997; Singh *et al*. 2001; Ulrich & Cerami 2001), or genetic variations affecting the amino acid sequence and thus changing the quantity of hydrogen bonds within the collagen molecules (Uzel & Buehler 2009) can also contribute to the variation of the Young's modulus. At the present stage of investigation we cannot exclude these factors. However, we suppose that the increase in collagen stiffness caused by the formation of AGEs with biological age is small because the reference sample was drawn from a volunteer of a similar biological age as the Iceman. Furthermore genetic level differences seem to be rather unlikely, because evolutionary changes occur over considerably longer time frames.

Our nanoindentation measurements in concert with Raman spectroscopy help to relate the mechanical properties and the molecular preservation of the collagen with the mummification process. This demonstrates the potential of non-invasive methods to access the mechanical properties and the structural preservation of ancient tissue. We presume that supplementary cross-links between the tropocollagen subfibrils reinforce their structure, making lateral displacement of the subfibrils more unlikely, thus increasing Young's modulus and the stability of the mummified collagen. Our data also picture the excellent degree of preservation of the Tyrolean Iceman and may be used as a reference to monitor the conservation of the mummy on a molecular level.

## 5. Conclusions

Skin tissue samples were extracted from three sites of the Iceman to examine the structural preservation of mummified type I collagen. The samples were processed to thin sections and analysed using AFM and Raman spectroscopy. Both methods indicate that the ultrastructure and molecular structure of the mummified collagen were preserved extremely well. Raman spectroscopy revealed spectra that were characteristic of type I collagen, and the amide I (1667 cm^−1^) and amide III (1245–1270 cm^−1^) bands indicate that the collagen molecules retained their helical conformation.

Examining single fibrils by nanoindentation demonstrated that Young's modulus of the mummified collagen was increased slightly over a recent sample. Although this stiffening might be supported by the effects of AGEs, genetic differences, or a very short period of UV irradiation of the fibrils, the most probable cause is dehydration. The loss of interstitial water resulted in a more densely packed structure of the fibrils and the generation of additional cross-links within the collagen. No evidence for collagen degradation was found that could have been caused by freeze–thaw cycles, micro-organisms or other biological influences.

Our results further show that the ultrastructure of the collagen fibrils remained unchanged for millennia owing to mummification by freeze-drying, and the enclosure of the body in glacier ice. Because of its low nutritional value, the dehydrated skin has maintained its protective function and prevented the connective tissue from decomposition. This result also shows the importance of dehydration for the mummification of connective tissue and supports the theory that the Iceman was covered by snow and ice immediately after his death (Bereuter *et al*. 1997; Rollo *et al*. 2000). Most probably, he was exposed to periodical cycles of thawing and freezing later on, which has resulted in advanced desiccation of the body.

## Appendix A

### (a) AFM nanoindentation

The measurement of the mechanical properties of collagen provides insight into its molecular preservation. To assess the tensile mechanical properties of these protein fibrils, optical tweezers (Sun *et al*. 2002), the AFM (Graham *et al*. 2004) or dedicated microelectromechanical systems (Shen *et al*. 2008) can be used. A compressive test by nanoindentation is required to measure the mechanical properties of fibrils that completely adhere to the substrate. The mechanical properties of collagen were examined using the NanoWizard-II AFM, operated with rectangular, aluminium-coated silicon cantilevers (NSC35-B, MikroMasch, Tallinn, Estonia). The nominal spring constant, *k*_c_, of the cantilevers was 14 N m^−1^. Before recording force–distance curves, topographic images of the samples were acquired to define adequate measurement regions. For imaging, we operated the AFM in intermittent contact mode. Subsequently, 10 force–distance curves were recorded at the centre region of the elevated banding structure of a single collagen fibril to avoid measurement errors arising from the topography (Domke & Radmacher 1998). Limiting the loading force to a maximum of 50 nN resulted in fibril indentations that were smaller than 2 nm. This corresponds to an indentation smaller than 6 per cent of the fibril height. Blunt tips that had radii 50 nm < *R* < 100 nm were favoured for the measurements. Each indentation curve was taken at a rate of 0.5 s for both the tip-approach and the tip-retraction (1024 data points). After a series of measurements, the samples were rescanned to assess plastic deformation of the fibrils. For further analysis, only force–distance curves that had been measured within the elastic regime were considered. More than six single fibrils and *n* ≥ 150 force–distance curves were analysed for the Iceman sample A and the recent sample. Young's modulus was determined from the curves using a Hertzian model (Hertz 1881). A spherical indenter geometry was assumed, because the indentation depth was restricted to less than 2 nm.

### (b) Young's modulus calculation

When modelling the collagen fibril as a cylinder with radius *R*_f_ (Tan & Lim 2005; Heim *et al*. 2006), penetrated by a spherical indenter with radius *R*_t_, Young's modulus (*E*) can be calculated as:

A1

where *F* is the applied force, **δ** is the indentation depth and **ν** is the Poisson ratio of the material. *R*_e_ is the equivalent radius for the spherical indenter in contact with the cylinder. This equivalent radius is defined as:

A2

The radius of the collagen fibril was measured as half the height of its highest point above the substrate. According to Hooke's law, the applied force is the product of the cantilever deflection (*d*) and its spring constant, *k*_c_.

A3

The indentation depth in equation (A 3) can be substituted by the difference of the z-piezo displacement (*z*) and the cantilever deflection, generating equation (A 4), where *d*_0_ and *z*_0_ are the zero deflection of the cantilever and the displacement of the z-piezo at the contact point (i.e. the point at which the tip begins to interact with the sample repulsively), respectively.

A4

For the calculations, the Poisson ratio was set to 0.5, assuming an incompressible material. The actual cantilever spring constant was determined using the quality factor calibration as described by Sader *et al*. (1999), and the tip radius was measured by imaging a calibration grating (TGT01, MikroMasch, Tallinn, Estonia). All measurements were performed in air at room temperature. Young's moduli were determined by fitting equation (A 4) to each force–distance curve (Stark *et al*. 1998). The data analysis was carried out using SPM Image Processing software (JPK, JPK Instruments, Berlin, Germany).
